# *In Vitro* Evaluation of Sunscreens: An Update for the Clinicians

**DOI:** 10.5402/2012/352135

**Published:** 2012-11-27

**Authors:** Maria Pelizzo, Edoardo Zattra, Piergiorgio Nicolosi, Andrea Peserico, Denis Garoli, Mauro Alaibac

**Affiliations:** ^1^Institutes for Photonics and Nanotechnology, National Research Council, Via Trasea 7, 35131 Padua, Italy; ^2^Dermatology Unit, University of Padua, Via Battisti 206, 35128 Padua, Italy

## Abstract

Topical sunscreens contain molecules or molecular complexes that can absorb, reflect, or scatter UV photons. Evaluation of the efficacy of sunscreen products has been made through the Sun Protection Factor (SPF), a mean of quantitatively assessing *in vivo* the degree of protection offered by sunscreen products against solar radiation. *In vivo* evaluation of SPF has several drawbacks. First of all, this evaluation method is expensive in terms of money and time. Moreover, it raises several ethical issues concerning the potential damage to skin volunteers. Several *in vitro* techniques have been developed, but at present there is no broadly accepted method. In this paper, we will discuss some of the recent advances concerning the *in vitro* evaluation of sunscreens which would be acceptable for replacing *in vivo* assays.

## 1. Introduction 

Light photons irradiating the earth consist of 56% of infrared light photons (wavelength 780–5000 nm) and 39% of visible light (400–780 nm). Ultraviolet radiations (UVR) are the 5% of the light photons irradiating the earth. The UVR reaching the earth's surface are UVB and UVA. UVB (290–320 nm) contributes for about 5% while UVA (320–400 nm) for about 95%. UVC (100–280 nm) are totally absorbed by atmospheric ozone. Sun is the main source of UVR, but artificial sources of UVR have been developed in the last decades. Skin is the organ most affected by environmental sunlight. Interaction between UVR and skin involves mutagenic lesions as well as indirect genotoxic effects mediated by oxidative stress [1]. It is well known that UVR can damage many skin molecules and structures, including DNA [1]. UVR can modify purines or pyrimidines, can disrupt the link between genes, or can even delete parts of the genome [2, 3]. All these damages are usually reversible, thanks to the DNA-repair mechanisms. Unfortunately, sometimes the repair mechanism fails and inability to further read and transcribe can occur, leading to cell death or abnormal behavior like hyperproliferation or malignant transformation [2]. 

## 2. Photoprotection from UV Damage

Photoprotection from UV damage is an essential prophylactic and therapeutic element, consisting of clothing and glasses and topical sunscreens or systemic agents. Topical sunscreens contain molecules or molecular complexes that can absorb, reflect, or scatter UV photons. Evaluation of the efficacy of sunscreen products has been made through the Sun Protection Factor (SPF) [4, 5], a mean of quantitatively assessing the degree of protection offered by sunscreen products against solar radiation. The SPF value offers no clear indication of the degree of protection against UVA1 (340–400 nm). It is based on an *in vivo* test that measures protection against sunburn or erythema, a biological response produced primarily by UVA2 (320–340 nm) and UVB (290–320 nm). Thus on August 2007, FDA US proposed to change SPF into “Sunburn Protection Factor,” but it was rejected and the FDA published its final rule in 2011 keeping the “Sun Protection Factor.”

## 3. *In vivo* Evaluation of Sunscreens

The SPF listed on sunscreen products is intended to communicate the amount of erythemal UVR attenuation. More particularly, numerical SPF theoretically tells the user that he or she is protected *X* times better than without sunscreen where *X* is the labelled SPF. SPF is calculated by dividing the minimal erythemal dose (MED) of protected skin for the MED dose of unprotected skin. MED is a measure of the amount of energy per unit area (J·cm^−2^) required to cause minimal erythema. Validation of SPF *in vivo* is made through an artificial source of UVR on human subjects. In Europe, the SPF is accepted if determined in at least 10 subjects [4]. *In vivo* evaluation of SPF has several drawbacks. First of all, this evaluation method is expensive in terms of money and time. Moreover, it raises several ethical issues concerning the potential damage to skin volunteers. Finally, it evaluates only the erythema which is caused by UVB and UVA-II, therefore the protection against the remaining UVA spectrum of UVR is not represented in the SPF value. The importance of adequate UVA protection is apparent with improved understanding that UVA may induce damages to cellular DNA via oxygen radical species as UVA energy interacts with endogenous photosensitizers [6]. *In vivo* evaluation of sunscreen's UVA protection requires high doses of UVA, thus being troublesome for economical and ethical issues. Three methods have been proposed for *in vivo* UVA protection evaluation: IPD (Immediate Pigment Darkening), PPD (Persistent Pigment Darkening), and UVA-PF (UVA Protection Factor). IPD and PPD are based, respectively, on immediate (seconds) or persistent (2–24 hours) pigmentation changes of the skin caused by UVA irradiation. UVA-PF is based on the minimal erythematous responses and persistent pigmentation caused by UVA. The IPD method determines the smallest dose required to produce darkening of the skin with a clearly defined margin, observed immediately after exposure. This reaction is transient and is secondary to the photooxidation of existing melanin in melanosomes. PPD uses, as the end-point, pigmentation, which is maximal at 2–4 h after exposure [7–13]. 

## 4. *In Vitro* Evaluation of Sunscreens

An *in vitro* SPF test method would be advantageous if it could generate results faster and cheaper. Furthermore, it could avoid the ethical concerns associated with *in vivo* testing. Several *in vitro* techniques have been developed, but at present there is no broadly accepted method. *In vitro* approaches generally consist of a film of sunscreen applied to an artificial test substrate and a spectrophotometer which analyzes the amount of UVR passing through the film of product. In the case of transmission spectroscopy, sunscreen is generally applied to a substrate and its spectral transmission measured prior to and after exposure to a UV source. Several factors influence the spectrophotometric analysis, notably the different compositions of filters, the quality of spectrophotometer, the type of the artificial test substrate, the amount of sunscreen applied on the substrate, and the spreading method. 

Photostability can be tested using an artificial UVR source (a solar simulator) by repeating the transmission measurements after expositing the sunscreen applied to the artificial substrate to this source. One of the most commonly used simulators is a Xenon Arc solar simulator. Such a simulator can produce light spectra meeting the SPF testing specifications set by the European Cosmetic Toiletry and Perfumery Association (“COLIPA”) and routinely used for *in vivo* SPF testing. A Xenon Arc solar simulator cuts off radiation at about 380 nm, meaning neither infrared nor visible radiation are emitted. In our laboratory we use an ORIEL 300 W full spectrum solar simulator. These solar simulators are accessorised with appropriate filters in order to discard the contribution of selected wavelengths. 

Several different artificial substrates have been used in this type of analysis. The test substrate must be as close as possible to the skin's physical characteristics. Substrates that are commonly used are Transpore, Vitro-Skin, Roughened Quartz Plate, polymethylmethacrylate (PMMA) plates, and PTFE (Teflon). Transpore (3M Company Health Care, ME, USA.) is a surgical tape. It has initially proposed as a readily available and inexpensive substrate, but now is not commonly used anymore. It is usually used as attached on a smooth quartz plate in order to get a hard support for the sunscreen appliance. Vitro-Skin (IMS Inc.) is a synthetic skin substrate that must be used following an exact hydration procedure. Published data [14] indicate that Vitro-Skin gives very good performances for sunscreen tests, even if the use of this material presents many disadvantages, notably a relatively high cost per sample, the need to hydrate the substrate starting the day before testing, a relatively short lifetime of the hydrated sample. Roughened Quartz is largely used because of its high-UV transmittance. Its main disadvantage is the high cost. In order to use again the same sample, a cleaning procedure can be followed. PMMA plates now appear to be the industry choice for *in vitro* testing in the UVA. It is very easy to handle and can be supplied with a reproducible roughness. PTFE has been extensively used for UV application due to its quite good transmittance and its Lambertian properties. Samples are treated with a blast sanding process in order to get a roughened surface. Biologically-derived substrates, like excised stratum corneum and excised human epidermis, have also been used but the outcome of the tests using these products was generally not reproducible [15]. 

At present, three *in vitro* methods for evaluation of protection from UVA are available, one from United States (FDA), one from European Union (COLIPA), and one from United Kingdom (Boots). The FDA proposal [16] measures the UV transmittance through a sunscreen film using the critical wavelength method. The critical wavelength is a measure of absorbance across the entire solar terrestrial UV spectrum (UVB and UVA radiation). The critical wavelength value for the test product is defined as that wavelength where the area under the absorbance spectrum for the irradiated product (obtained using the method described above) from 290 nm to critical wavelength is 90% of the integral of the absorbance spectrum from 290 nm to 400 nm. Sunscreen products offering primarily UVB protection would have a critical wavelength less than 320 nm, whereas those providing both UVB and UVA protection would have critical wavelengths between 320 and 400 nm. FDA requires that sunscreen products have a critical wavelength of at least 370 nm (the mean value must be equal to or greater than 370 nm) to be labeled as providing “broad spectrum” UVA and UVB protection. UV radiation in the range of 370–400 nm is not very harmful based on the available action spectra for sunburn and skin cancer. Most of the harmful effects from the sun are caused by UV radiation in the range of 290–370 nm. The SPF is an index which is not sensitive above 340 nm thus it is not a good measure for broad spectrum protection. The critical wavelength (breadth UVB and UVA protection) coupled with the SPF value does not provide a complete measure of broad spectrum protection provided by a sunscreen product. It has been demonstrated that only UVAPF is able to measure the amplitude of UVA protection and that values obtained with UVAPF for critical wavelength at least 370 nm are significantly different from those obtained with SPF [17]. This is the reason why the European Commission recommends since 2006 for all sunscreen products a ratio SPF/UVAPF ≤3 and a critical wavelength at least 370 nm in order to have both the sufficient level and the broadness [18]. The Colipa *in Vitro* Method [19] first calculates *in vitro* the UVA protection factor (UVA-PF). The *in vivo* UVA-PF derived from the PPD method have been shown to correlate well with the *in vitro* UVA-PF method based on an assessment of the UV transmittance of a thin film of sunscreen sample spread on a PMMA roughened substrate after exposure to a controlled dose of UV radiation from a defined UV source. Second, the method also provides a means of calculating critical wavelength values. The final critical wavelength value for each tested sunscreen product is the mean of values recorded for each measured, irradiated, and product-treated PMMA plate. The UK method, also called Boots star rating system [20], also measures the UV transmittance through a sunscreen film. The substrate for measurement is abraided PMMA plates. The ratio between the mean UVA and UVB absorbance measured before and after irradiation of the sunscreen products is calculated. The final outcome of this evaluation can be no stars, 3, 4, or 5 stars. *In vitro* characterization based on transmission measurements is now the preferred method to assess UVA protection and *in vivo* test.

*In vitro* evaluation of UVB protection (SPF *in vitro*) has been first proposed by Diffey in 1989 [21]. At present there are several SPF *in vitro* methods which are only used for products development and screening. There is no official, standardized, harmonized published method accepted for SPF labeling by authorities. Among these methods, the National Institute of Public Health (NIPH) method [22], the Výzkumný ústav organických syntéz (VUOS) (Research Institute for Organic Syntheses) method [23], a method using artificial substrates and a novel pseudodouble beam mode of operation of a standard double beam UV spectrophotometer [24], and the SONING method [25]. NIPH method [22] measures the attenuation of UVB intensity on a defined layer of a sunscreen product, using an UVA/UVB source, a sheet of Mikelanta covering paper with 2 mg/cm^2^ of product applied with an ungloved finger, and a radiometer for UVB radiation intensity measurement. The VUOS method [23] uses a surgical tape posed on a quartz layer with 1.2 mg/cm^2^ of product applied; a spectrophotometer then measures the transmittance and the SPF is calculated. The method described by Bleasel and Aldous [24] uses diffusing plates made of quartz with Transpore adhesive tape or human stratum corneum obtained from a skin surface biopsy as substrate. A pseudodouble beam mode of operation of a standard double beam UV spectrophotometer is utilized, greatly increasing the effective linear range of the detector response of the spectrophotometer. This method can be used with both high and low SPF value sunscreens. SONING method [25] uses a UVB source, a sheet of tracing paper with a 2 mg/cm^2^ of the sunscreen product, and an electronic UVB intensity meter. Since its introduction in 2001 the *in vitro* test method on roughened PMMA plates is widely used [26]. However recently it has been demonstrated that relative indices based on absorbance such as the UVA/UVB ratio and critical wavelength and indices based on protection factors such as the SPF/UVAPF ratio show a significant variation as a function of roughness. Absolute indices like the *in vitro* SPF and UVAPF are also very sensitive to roughness variation and this explains the lack of reproducibility often reported for the *in vitro* SPF [27].

In a recent investigation [28] we have correlated the correspondence between *in vitro* SPF data and values reported by the manufacturers. We applied the product on PTFE plates in standardized condition and then we measured the absorbance/transmittance with a spectrophotometer. The results showed that there is a good correlation between the *in vitro* SPF determined by the present method and the SPF reported by the manufacturer. This seems to be particularly true when a small quantity of product is applied on the substrate, such as the case of 0.65 mg/cm^2^. Furthermore, we performed photostability tests by irradiating the products with different wave-bands. This method has never been used before and our results demonstrate that both UV and IR wavelengths can affect the absorbance characteristics of the sunscreen. 

At present the FDA is not replacing the *in vivo* SPF test with an *in vitro* SPF test [16], since one of the limitations of an *in vitro* test is the lack of data on the performance characteristics of *in vitro* test substrates, such as quartz or artificial skin. FDA, in the 2007 rule [29], stated that data failed to show that a substrate could effectively simulate the complex features of human skin. In the new 2011 rule [16] FDA decided to confirm the exclusion of an *in vitro* test, due to the lack of new data to validate *in vitro* tests.

## 5. Conclusions 

Because of the use of high sun protection factor sunscreens, high level of UV doses must be utilized to assess their protection factor; consequently, it would be wise to replace human testing with *in vitro* approaches. At present, however, there is no standardized *in vitro* method accepted for SPF labeling by authorities. Only research methods have been proposed. More studies are needed to validate and standardize methods for measuring the *in vitro* protection factors in order to obtain meaningful information for physicians and consumers. 
